# A Method for Determining the Minimum Thickness of the Cut Layer in Precision Milling

**DOI:** 10.3390/ma18010189

**Published:** 2025-01-04

**Authors:** Lukasz Nowakowski, Mateusz Bronis, Slawomir Blasiak, Michal Skrzyniarz

**Affiliations:** Department of Machine Design and Manufacturing Engineering, Kielce University of Technology, al. Tysiaclecia Panstwa Polskiego 7, 25-314 Kielce, Poland; lukasn@tu.kielce.pl (L.N.); sblasiak@tu.kielce.pl (S.B.); mskrzyniarz@tu.kielce.pl (M.S.)

**Keywords:** minimum chip thickness, milling process, milling, anova

## Abstract

The minimum cutting thickness is a key value in machining processes, as below this value the material will only undergo elastic and plastic deformation without chip removal. Existing measurement methods require time-consuming preparation and complicated procedures. This work focuses on the development of a new, simplified method for determining the minimum cutting thickness (h_min_) using a contact profilometer that can be used in industry. The use of the contact measurement method has made it possible to directly determine the value of the h_min_ parameter, to determine the length of the characteristic zones of interaction of the tool with the surface of the specimen, and to measure the angle of inclination of the working plane of the specimen. Measurement using a profilometer allows for the obtainment of results with high resolution, which greatly facilitates the identification of zones of tool interaction with the workpiece material during the cutting test and reduces the value of the measurement error. The proposed method simplifies the specimen preparation process by using rectangular specimens positioned on an inclined plane, which allows the depth of the cut to be varied smoothly. This paper presents experimental results and statistical analysis. Tests were carried out on C45 steel, and an ANOVA analysis was carried out to evaluate the effect of the grinding parameters on the h_min_ parameter. It was found that the feed rate had the largest effect on h_min_ (93%), while cutting speed had a smaller effect. A mathematical model was developed to predict values based on selected technological parameters such as cutting speed and feed per tooth.

## 1. Introduction

The finishing process is carried out with small thicknesses of the machined layer, so it is necessary to know the minimum thickness of the machined layer (h_min_), beyond which the phenomenon of decohesion—i.e., the start of cutting—occurs [1,2,3,4,5].

Under actual machining conditions, the cutting edge of the milling head insert is not perfect; it has some cavities and a roundness with edge radius r_n_ due to its manufacturing technology [6,7,8,9]. During fine machining, it is particularly important to take into account the actual shape of the cutting edge. The quantity defining the start of material separation is the minimum thickness of the machined layer (h_min_). This is the smallest layer of material that can be removed under given machining conditions. Depending on the thickness of the machined layer in the cutting zone, three stages of the interaction of the cutting edge with the material to be machined can be distinguished [10,11,12,13,14,15,16]:

I When a_p_ < h_min_: elastic deformation and plastic kneading of the workpiece material occur;
II When a_p_ ≈ h_min_: elastic–plastic deformation occurs with partial cutting of the workpiece material;
III When a_p_ > h_min_: start of chip removal.

Analysis of the literature revealed several relationships that were developed to estimate h_min_ values. A summary of the relationships is given in Table 1.

**Table 1:** Summary of formulae for calculating the value of the minimum thickness of the cut layer.

No.	Model		Authors
(1)	$h_{\min}=r_{n}\left(1-\cos\theta \right)$	[17,18]	Malekian and Lee
(2)	$h_{\min}=k\cdot r_{n}$	[19]	Kawalec
(3)	$h_{\min}=f_{z}\cdot \sin\theta$	[20]	Sai
(4)	$h_{\min}\geq 0.5\;r_{n}\left[1-\frac{2\;\tau_{a}}{\sigma_{ch}}\right]$	[16]	Grzesik
(5)	$h_{\min}=\Psi \;\;\frac{E}{H}\;\;{\left(\frac{K_{IC}}{H}\right)}^{2}$	[21]	Blake
(6)	$h_{\min}=r_{e}\left(1-\frac{1}{\sqrt{1+\cos\theta^{2}}}\right)$	[22]	Li
(7)	$h_{\min}=r_{n}-\left(\sin\left(90^{o}-\Psi_{kryri}\right)r_{n}\right)$	[23]	Wojciechowski
(8)	$h_{\min}=r_{n}\left(1-\frac{\frac{a_{f}}{a_{c}}}{\sqrt{1+{\left(\frac{a_{f}}{a_{c}}\right)}^{2}}}\right)$	[24]	Storch

The most common definition found in the literature to describe the minimum thickness of the machined layer is the relationship of the minimum thickness of the machined layer to the edge radius r_n_ [15,17,18,19,21,22,23,24]. The stagnation angle is another important factor taken into account in the equations that determine the value of the h_min_ parameter [17,18,20,22].

**Figure 1:**
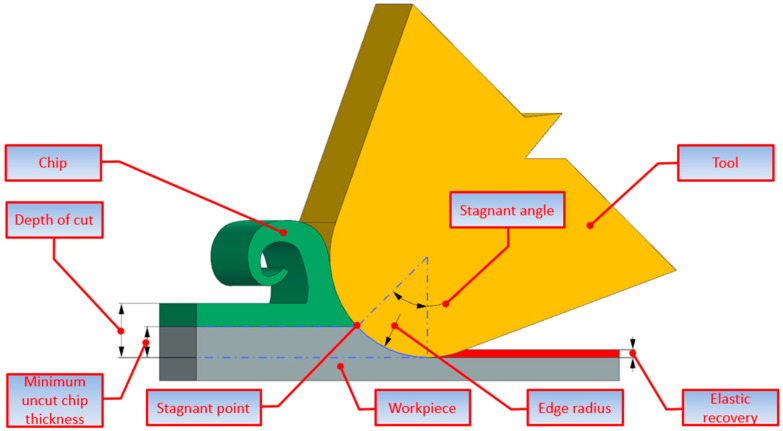
Material flow around the edge of the tool with an edge radius r_n_.

A diagram of the material flow around the edge of a tool with edge radius r_n_ is shown in Figure 1. The stagnation point A marks the division into two zones above and below the point. Below the stagnation point A, the material flows down the edge of the tooth without forming a chip, a phenomenon called furrowing, where elastic–plastic deformation occurs without material removal. Above the stagnation point, the material flows and is removed in the form of a chip. The location of the stagnation point on the edge radius is defined by the stagnation angle θ. The stagnation angle θ and the negative effective angle of friction α = π/2 − θ determine the minimum thickness of the cut layer in relation (1) in Table 1.

Kawalec proposed relation (2) taking into account the proportionality factor in the relation h_min_ = k·r_n_, which depends on the friction conditions between the tool and the machined material. The coefficient may take values in the range of 0.1–1 [19]. A common relation based on the tool path is relation (3) [20]. The tool path is represented by the feed per tooth and the instantaneous angular position of the tool. Regrettably, this is a simplified model that assumes that the path is circular, the milling process is ideal, the tool is free of manufacturing errors, and the machine has no geometry errors and is rigid.

In his work [16], Grzesik developed relation (4) for the minimum thickness of the scratch layer using the molecular–mechanical theory of dry friction. Determination of the h_min_ value requires knowledge of the equation of the strengthening curve and experimental determination of the equivalent strain and temperature in the adhesive joint.

The critical value of the h_min_ parameter is defined by relation (5) in Table 1, and relation (5) was developed by Blake and Scattergood [21]. It takes into account (K_IC_)—fracture toughness, (E)—elastic modulus, (H)—Knoop hardness, and (y)—a dimensionless constant depending on the cavity geometry. When analyzing the relationships and methods developed for determining the minimal thickness of the cutting layer, it was found that most of them take into account only the roundness process of the cutting edge. Others require the knowledge of many complicated relations or the experimental determination of constants. The proposed relationships only allow for the determination of limiting or critical values without defining their variation with the cutting conditions and the workpiece material.

**Figure 2:**
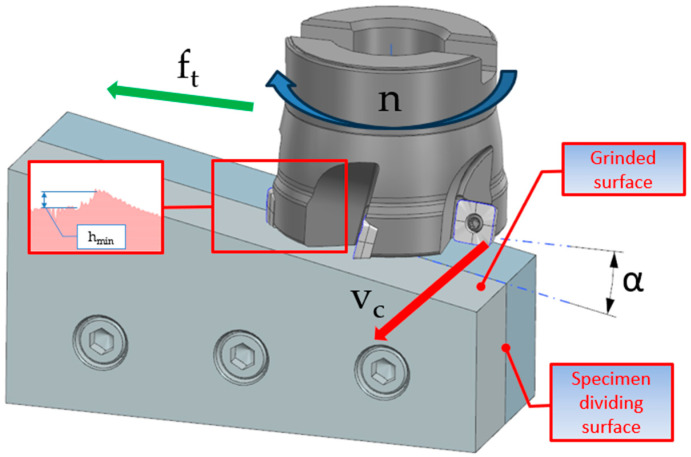
Method of determining h_min_ according to the patent.

In addition to the mathematical models used to predict the minimum layer thickness, methods were developed for the practical determination of h_min_ (Figure 2), which consist of conducting cutting tests with varying cut layer thicknesses. The variation in cut thickness was achieved in two ways: stepwise by gradually changing the depth of the cut or smoothly by inclining the working surface of the specimen relative to the direction of tool displacement. The stepwise variation of the machining thickness makes it impossible to smoothly determine the characteristic zones of material decohesion initiation. Inclination of the specimen’s working surface in relation to the direction of tool displacement gives a linear relationship for the change in thickness of the cut layer and at the same time makes it possible to determine the characteristic zones of initiation of the material separation phenomenon. Determination of the lengths of the characteristic cutting zones is carried out using a shop microscope on a measuring field. The measuring field requires the metallographic impression to be made by successive cutting, grinding, and polishing operations, which distort the image of the phenomenon, making it the result of auxiliary machining processes. In another method patented by Harasymowicz [25], a modification of the sample preparation procedure was introduced in order to eliminate errors introduced by the cutting and grinding of the deposit on the measured surface. For this purpose, prior to the cutting test, the specimen is cut in a plane perpendicular to the direction of the cutting speed, the split surfaces are machined with a high-precision finish, and then the specimen parts are rigidly joined together. After the cutting test, the specimen is split, and the edge fragment between the parting surface and the specimen working surface is analyzed using a microscope.

Li and co-authors [22] proposed relation (6). It can be measured with a force meter in the X and Y directions, which are converted into the tangential milling force F_t_ and radial milling force F_c_ of the tool, and the relationship between the milling forces in each direction is expressed as Equation (9)

(9) $$\frac{F_{t}}{F_{c}}\left(\theta =\frac{\pi }{2}\right)=\frac{F_{x}\sin\theta +F_{y}\cos\theta }{F_{x}\cos\theta -F_{y}\cos\theta }=-\frac{F_{x}}{F_{y}}$$

Wojciechowski developed relation (10). It was based on an analysis of the tangential forces acting on a rounded cutting edge. The force models were expressed as a linear function of the thickness of the cutting layer. This made it possible to determine the stagnation angle $\beta_{kr}$ according to relation (10).

(10) $$\beta_{kr}=arcctg\left(\frac{a_{f}}{a_{c}}\right)$$

where a_f_ and a_c_ are directional coefficients in the linear regression equations for the feed force and cutting force.

The methods presented above for determining the minimum thickness of the cut layer require complicated and time-consuming preparation of the measurement sample. The procedure for preparing such specimens requires cutting the specimen according to a plane perpendicular to the direction of the cutting speed, making a rake on the post-measurement surface, and rigidly connecting the parts of the cut specimen to each other using locating pins and screws. A gradient of the specimen’s working surface relative to the direction of tool displacement must then be obtained on the specimen by means of a grinding operation. The microscope method of measurement requires a great deal of experience on the part of the measurer and can be subject to error due to the incorrect determination of characteristic areas. Such a complicated sample preparation procedure makes it very difficult to use this method more widely, e.g., in industry.

The aim of this research was to develop an easy and user-friendly method for determining the minimum thickness of a machined layer that can be applied in industrial practice. The method is based on the measurement of the minimum cut thickness using a contact profilometer, which replaced the inaccurate microscope. This paper presents the method illustrated and the results developed during an experiment on C45 steel. When analyzing the results, an ANOVA statistical analysis was used to determine the influence of individual input parameters on the output parameter, minimum cut thickness. The equation defining the prediction of individual results is also presented and compared with the actual results. The given prediction model h_min_ was then simulated in relation to the technological parameters.

## 2. Materials and Methods

Specimens with an octagonal cross section of 55.5 mm in height and 60 mm in length made of C45 steel were used for cutting tests. C45 steel is a steel grade that is characterized by difficulty of welding and ease of machining. It has an increased tendency to stick together, forming growths on the tooths. The steel has poor machinability, resulting in rough surfaces. It is used for medium-duty machine elements such as spindles, axles, crankshafts, unhardened gears, electric motor shafts, ordinary knives, corkscrews, wheel hubs, discs, rods, rollers, and pump rotors. The products can be surface hardened to a hardness of up to 50–60 HRC. The properties and chemical composition of the tested steel are given in Table 2 and Table 3, respectively.

**Figure 3:**
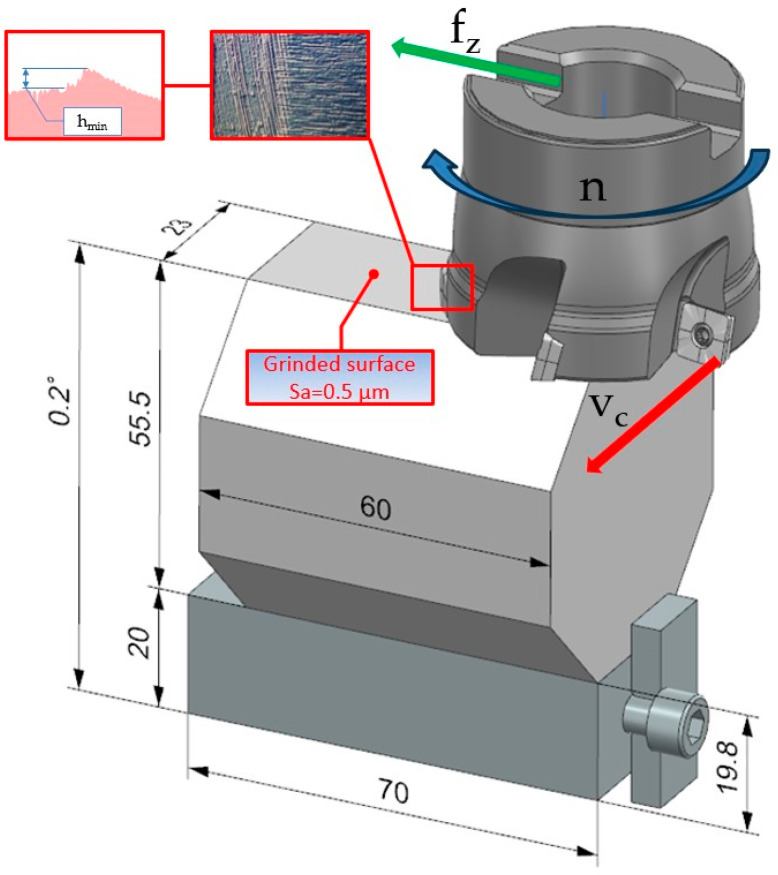
New method of determining h_min_.

The method developed is intended to make it easier, quicker, and more accurate to determine the minimum thickness of the machined layer. To this end, it was decided to simplify the design of the specimen used during testing and to change the method of measuring the length of the characteristic zones of tool interaction with the workpiece material. As in previous methods, it was decided to incline the working surface of the specimen relative to the direction of tool displacement in order to obtain a smooth transition between the zones of initiation of the material decohesion phenomenon. In current methods, the inclination of the specimen’s working surface relative to the direction of tool displacement is achieved by means of a grinding operation, which consists of making a specimen that has its working surface inclined at an angle. This is a complicated and time-consuming machining process that requires the use of special grinders. In the newly developed method, a uniform rectangular specimen was chosen, whose surfaces can be easily prepared on a surface grinder. In the initial stages of the research, the inclination of the specimen’s working surface was achieved by placing a slotted plate of a specified thickness under the specimen on one side and rotating the inclined specimen with the jaws in a vice. The problem was that the solution used was characterized by poor clamping repeatability and varying specimen tilt angles. In this case, it was decided to change the clamping method and use the solution shown in Figure 3, where the slot gauge sheets were replaced by an accurately made inclined plane with a known inclination angle α = 0.2°, equipped with a special bumper for precise positioning of the plane in the vice and accurate and repeatable positioning of the specimen on the plane.

**Figure 4:**
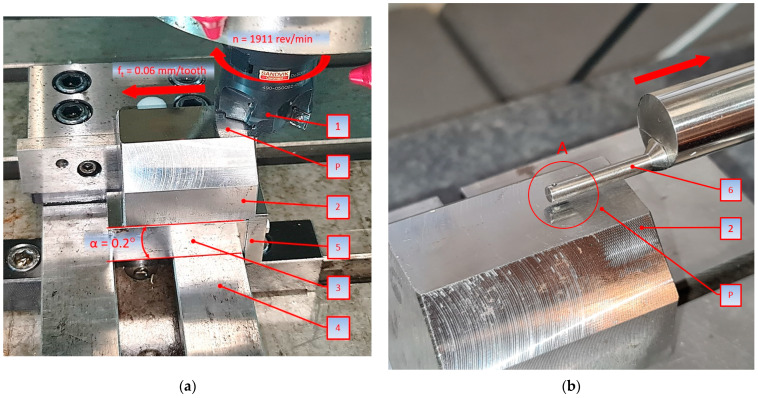
Method of mounting and measuring the specimen for determining the minimum thickness of the cut layer. (**a**) Positioning the specimen and tool in the side view and (**b**) measuring the minimum thickness of the cut layer, where 1 is the milling head; 2 is therectangular specimen; 3 is the inclined plane; 4 is the vise; 5 is the limiter; 6 is the profilometer; A is the measuring head and P is the working surface.

A full understanding of the measurement method will be provided by a description of the exemplary tests performed, the steps of which will be explained on the basis of Figure 4, which shows the positioning of the sample and tool in side view when the minimum thickness of the machined layer is tested using the milling head 1, Figure 4a, while Figure 4b shows an example of the measurement.

**Figure 5:**
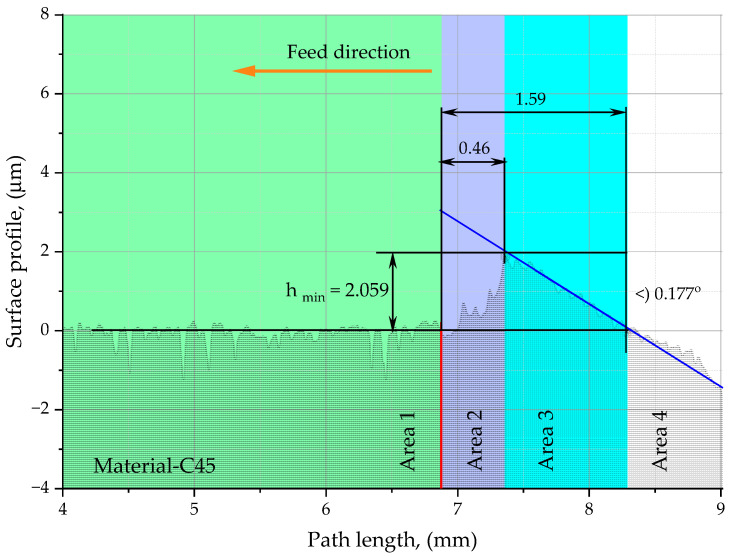
Surface profile obtained by measuring on the profilometer TOPO 01P with individual zones of the interaction of the 490-050Q22-08M CoroMill cutter tool with the workpiece material. Cutting parameters: v_c_ = 300 m/min, and feed per tooth f_z_ = 0.06 mm/tooth.

The working surface P of the octagonal specimen 2 was machined on a JOTES SPC 20 A surface grinder (Grinder Factory ‘Jotes’, Łódź, Poland), so that the surface roughness of the specimen (Sa = 0.5 µm) is one order lower than the surface roughness obtained during the cutting test. In order to obtain a linear change in the thickness of the cut layer, the octagonal sample 2 was placed on an inclined support 3 with an angle of α = 0.2°. The inclined support 3 is equipped with a stop 5, which ensures repeatability of the positioning of the specimen 2 on the prism 3 and the repeatability of the positioning of the prism 3 with respect to the chuck 4. Cutting tests were conducted for the 490-050Q22-08M insert mounted in a Sandvik Coromant 490-050Q22-08M CoroMill cutter 1 attached to an AVIA VMC 800 vertical milling center (Precision Machine Tools Factory “AVIA” S.A., Warsaw, Poland). The example specimen work surface shown in Figure 4 was machined with a cutting speed of v_c_ = 300 m/min and feed per tooth f_z_ = 0.06 mm/tooth. After the cutting test, the sample 2 was placed on the Table 7 of the TOPO 01P profilometer (Institute of Advanced Manufacturing Technology, Krakow, Poland), and the machined surface P was measured at the starting point of the cutting process A (Figure 4b) using the non-slip head 6 (r_rip_ = 2 mm, α = 90°) of the profilometer, obtaining the surface profile shown in Figure 5 using the ‘Topography TOPO 01 v1′ software (Institute of Advanced Manufacturing Technology, Poland). Detail A in Figure 5 shows the surface profile obtained from the profilometer with the individual zones of influence of the tool on the workpiece material.

The profile shown in Figure 5 shows the machining marks to identify the zones of chip formation and to determine h_min_, which, for example, in the machining of carbon steels is approximately 0.002 mm.

Area 4 is the section that has been ground and ends at the level of the milled sample profile. The h_min_ parameter is measured from this point. Area 3 defines the area where the tool made contact with the material but was unable to form a chip due to the shallow depth of the cut. Area 2 corresponds to the start of material removal in the form of a chip. Area 1 represents the regular profile formed as a result of milling.

**Figure 6:**
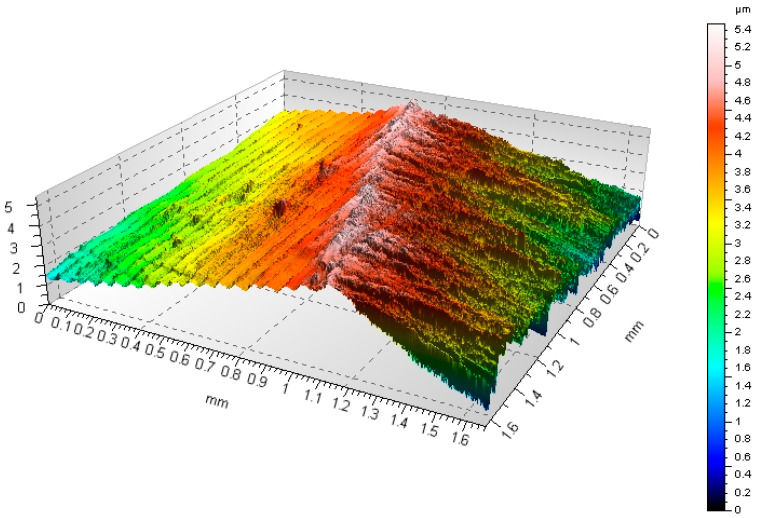
Isometric views of the profile obtained by measuring on the profilometer TOPO 01P Taylor Hobson Talysurf CCI—Lite Non-Contact 3D optical profiler.

The presented measurement method provides a quick, easy, and highly accurate measurement of the minimum thickness of the machined layer and eliminates the labor-intensive and cumbersome operation of cutting the specimen, making a deposit, and twisting and grinding against the surface of the specimen. It also replaces the low-accuracy, low-resolution, and depthless measurement using a microscope with a contact profilometer. The method makes it possible to faithfully diagnose the phenomena that occur when initiating or sustaining a cutting process. The developed method makes it possible to determine the influence of various factors such as the physico-chemical properties of the workpiece material, tool geometry, and machining conditions on the value of the minimum thickness of the machined layer. By standardizing the geometry of the cutting edge and the sample, the method makes it possible to evaluate and compare the cutting capacity of cutting tools, especially for machining with small cutting layer thicknesses, in relation to different machining parameters. The 3D isometric image (Figure 6) was observed using a Taylor-Hobson Talysurf CCI-Lite Non-Contact 3D optical profilometer (Taylor-Hobson Ltd., Leicester, UK) at a magnification of ×20. This area allowed the shape of the 2D profiles of the observed zone to be assessed. The examined area consisted of 1024 individual 2D profiles, corresponding to an area of 0.8 mm × 0.8 mm.

## 3. Results

The subject of this study is to determine the influence of selected machining factors on the minimum thickness of the cut layer (h_min_) during face milling of C45 steel.

On the basis of literature studies and previous in-house research, it was considered that a static determined single-factor selection PS/DS-U program was the most suitable experimental test program. The variable factors were feed per tooth f_z_ and cutting speed v_c_. The machining parameters were selected according to the manufacturer’s recommendations, taking into account the material to be machined. Results in Table 4 were obtained by measuring the profiles of the working surfaces of the samples for the determination of the h_min_ parameter on a TOPO 01P contact profilometer.

The starting material for the test specimens was round bars with a diameter of ϕ60 mm and a length of 1000 mm.

The main consideration in determining the shape of the specimen was how it would be clamped on the machine tool; in the case of milling machines, a fast and accurate clamping system is a precision vise. A Bison Bial 6620 precision vise (Bison S.A., Białystok, Poland) was used to clamp the specimens during all cutting tests.

Another factor that determines the shape of the experimental specimen is the number of cutting tests that will be carried out on a single sample. The preliminary test plan shows that at least four different cutting tests will be carried out on a single specimen. Taking this into account, it was decided to produce specimens with an octagonal cross-section. This cross-section of the specimen allows problem-free clamping in a vice, as it has four pairs of parallel faces, allows the specimens to be clamped in a four-jaw chuck, and also makes it possible to measure the specimens on various gauges without using grips. A final advantage of this specimen geometry is the eight surfaces on which cutting tests can be carried out without having to re-prepare the specimen surface.

In order to illustrate the test results, graphs of the variation in the value of the minimum thickness of the cut layer h_min_ as a function of feed per tooth and cutting speed were prepared.

**Figure 7:**
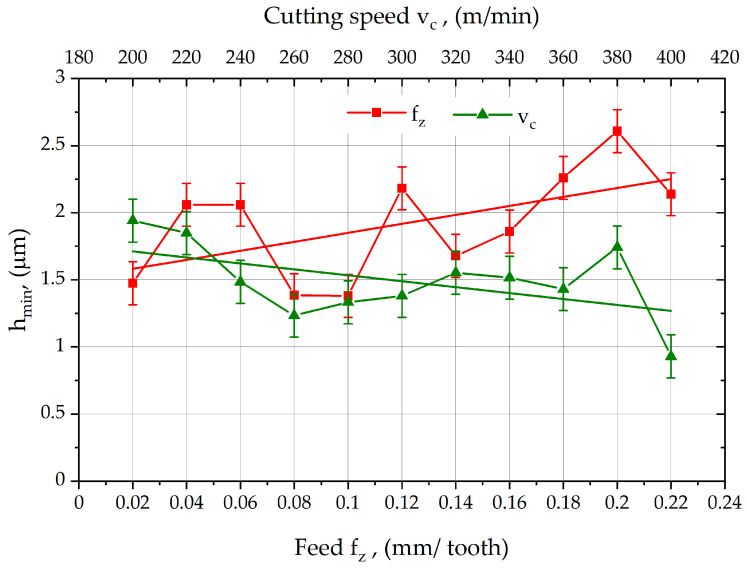
Results of measurements of the h_min_ parameter values for C45 steel.

The graph in Figure 7 shows the influence of selected cutting parameters on the value of the h_min_ parameter during face milling of C45 steel. When analyzing the effect of the feed rate f_z_ on h_min_, it was observed that the values of h_min_ oscillate around 1.95 µm for feed rates of 0.02 ÷ 0.14 mm/tooth, before increasing to 2.6 µm for the feed rate of 0.2 mm/tooth. In order to determine the effect of f_z_ on h_min_, a trend line was introduced on the graph, which shows that as the feed per tooth increases, the value of the h_min_ parameter shows an increasing trend.

When analyzing Figure 7 showing the effect of cutting speed on the value of the minimum thickness parameter of the machined layer h_min_, it was found that, in the initial phase, an increase in the cutting speed v_c_ resulted in a decrease in the value of the parameter h_min_. A slight increase in the h_min_ parameter was again observed once the cutting speed exceeded 260 m/min, and this slight upward trend continued up to a cutting speed of 320 m/min. Further increases in the cutting speed resulted in a decrease in h_min_ until v_c_ = 380 m/min, where an increase in h_min_ occurred, before decreasing again. The overall effect of cutting speed v_c_ on the value of the minimum cut layer thickness parameter h_min_ is illustrated by the decreasing trend line introduced in the graph.

For C45 steel, an unfavorable upward trend in the h_min_ parameter was observed with increasing feed per tooth. This means that when designing finishing processes, we need to increase the thickness of the cutting layer to ensure material separation and avoid elastic deformation, plastic kneading, and elastic–plastic deformation with partial cutting of the workpiece material. Increasing the cutting speed resulted in a downward trend in the h_min_ parameter for C45 steel. When planning the finishing process, we need to increase the cutting speed to reduce the value of the removed layer of material.

Most of the developed relationships and methods for determining the minimum cutting layer thickness take into account the cutting-edge radius. The remaining relationships require the knowledge of many complex dependencies or the experimental determination of constants and only allow for the determination of threshold or critical values of the h_min_ parameter without defining its variability depending on the cutting conditions and the type of material being machined. Practical methods for determining the minimum thickness of the cut layer require complex and time-consuming sample preparation. The h_min_ measurement is carried out on optical instruments, which requires considerable experience on the part of the measurer in order to correctly interpret the individual zones of tool–material interaction during the initiation of the cutting process. The developed method allows for easier and more accurate determination of the minimum thickness of the cut layer (h_min_) by simplifying the geometry and procedure for preparing the sample for testing and by using a high-resolution measuring instrument. The use of the contact method allows for both direct and indirect determination of the minimum thickness of the cut layer by measuring the angle of inclination of the working surface of the sample. The high resolution of the measuring system facilitates the interpretation and identification of the zones of interaction between the tool and the surface of the machined specimen, making it easy to measure their length.

It is possible to identify characteristic areas of tool–material interaction based on surface microhardness measurements. Unfortunately, this is a very labor-intensive process and requires advanced measuring equipment and considerable experience on the part of the person performing the measurement. The results obtained can be used in models for predicting the roughness of milled surfaces, selecting the material allowance for finishing operations and determining the depth of cut for high-speed machining. When analyzing the developed relationships and methods for determining the minimum cutting layer thickness, it was found that most of them only consider the cutting-edge radius. The remaining methods require knowledge of many complex dependencies or experimental determination of constants. The proposed relationships only allow for the determination of threshold or critical values without defining their variability depending on the cutting conditions and the type of material machined. The method developed by the authors to determine the minimum thickness of the cut layer is fast and not labor intensive, making it suitable for industrial use.

### 3.1. Analysis ANOVA

This study applied the Taguchi Genichi method used to assess the quality of goods produced in Japan. For this purpose, an L21 orthogonal array was defined to include all the data presented in Table 4. The total degrees of freedom of the input effects were 20. The statistical analysis used a 95% confidence level at the 5% significance level. The analysis can be assumed to be significant if the *p*-values are less than 0.05. A polynomial model (otherwise known as a non-linear linearized model) presented as Equation (11) was chosen to determine the predictive model of the h_min_ parameter. In a polynomial model, the independent variables can occur in higher powers (square, third power, etc.).

(11) $$Y=b_{0}+b_{1}X_{1}+b_{2}X_{2}+b_{3}{X_{1}}^{2}+b_{4}{X_{2}}^{2}$$

where *Y* is the estimated response, $X_{1}$ and $X_{2}$ are process input parameters, $b_{0}$ is the free expression, and $b_{1}$, $b_{2}$, $b_{3}$, and $b_{4}$ are coefficients.

In Table 5, the SS and MS values were calculated to determine the F value. Based on this, the significance of a given analysis was read from the tables.

From the analysis, it can be concluded that the mathematical model built here is significant (*p* < 0.05). The minimum thickness of the machined layer is most influenced by feed rate per tooth, at 93%, with the remainder attributable to the cutting speed. Using Equation (11), the developed mathematical model of h_min_ is presented below as Equation (12).

(12) $$h_{\min}=1.51387+0.00463\cdot v_{c}-1.14154\cdot 10^{\left(-5\right)}{v_{c}}^{2}-7.02508\cdot f_{z}+44.51246\cdot {f_{z}}^{2}$$

**Figure 8:**
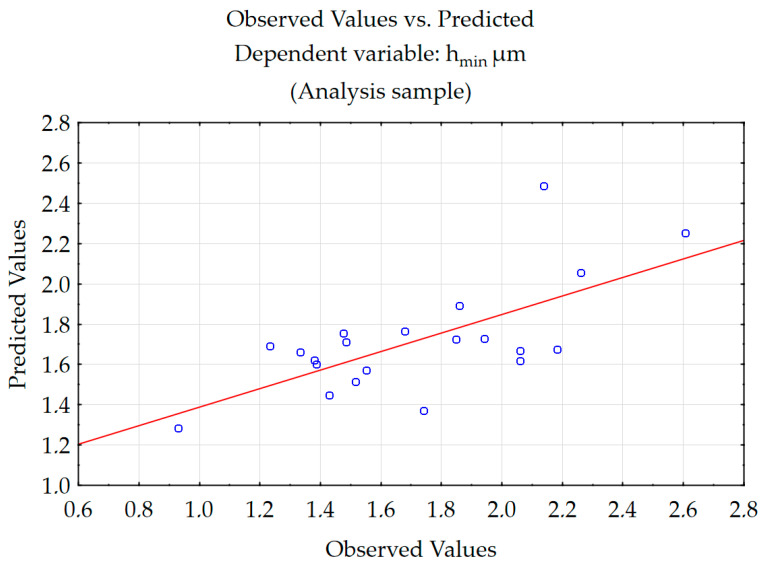
Comparison of experimental values with predictive values for h_min_.

The experimental and predicted values are shown in Figure 8. As can be seen, the values are in good correlation. The root mean square error (RMSE) was 0.247 µm.

**Figure 9:**
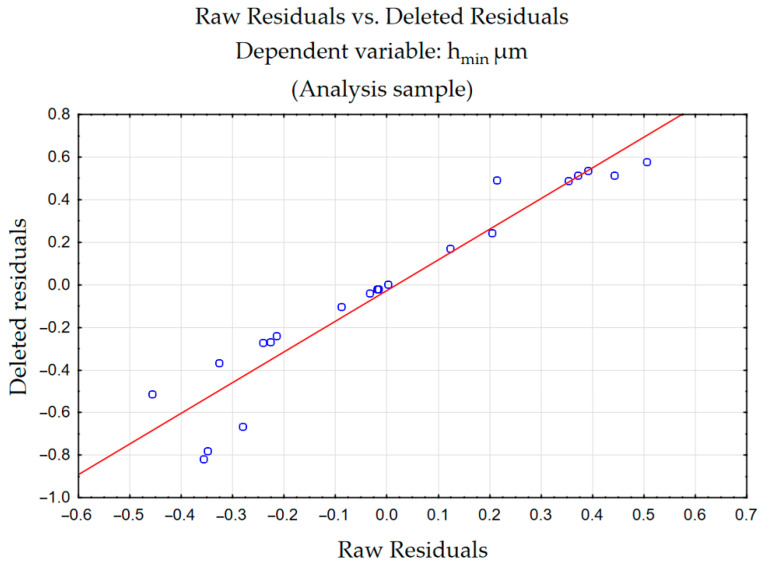
Normal probability plot for h_min_.

Figure 9 shows the plots of the residuals. It shows that the assumption of normality is met due to the small distance of the points placed relative to the line.

### 3.2. Simulation Studies of a Predictive Model for Minimum Cut Layer Thickness

**Figure 10:**
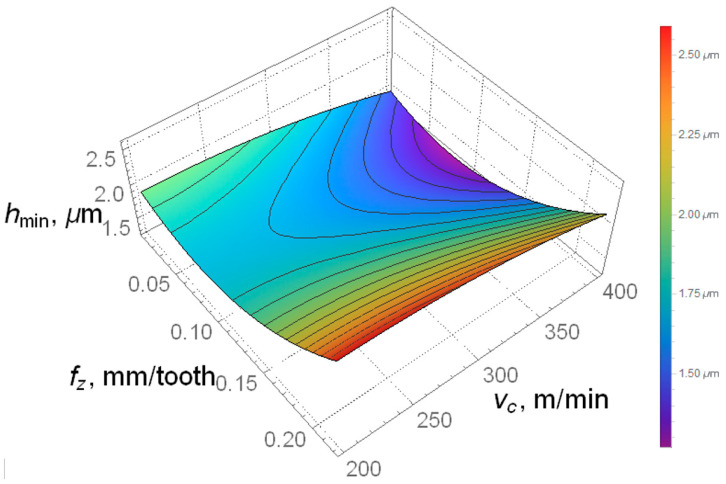
Simulation study of the built predictive model of minimum cut layer thickness.

Equation (12) was used to clearly illustrate the minimum thickness of the machined layer in order to more accurately depict the aforementioned parameter. Figure 10 shows that the smallest minimum thickness of the machined layer was obtained for the following technological parameters f_z_ in the range from 0.05 to 0.1 mm/tooth and the highest tested value of the cutting speed v_c_ = 400 m/min.

The highest value of the minimum thickness of the machined layer was obtained for the highest tested feed per tooth f_z_ = 0.22 mm/tooth and a cutting speed in the range of 200 to 280 m/min. It was observed that an increase in cutting speed reduces the minimum thickness of the machined layer in C45 steel.

## 4. Conclusions

The aim of this study was to design a methodology and carry out measurements of the minimum thickness of the machined layer and to determine the influence of the technological parameters of the cutting process on the minimum thickness of the machined layer.

The main conclusions of the above research are as follows:

- The developed method of measuring the h_min_ parameter allows for easier and more accurate determination of its value due to simplifying the geometry and procedure for preparing the sample for testing and the use of a high-resolution measuring instrument;
- Using the contact method, it was possible to directly measure the value of the h_min_ parameter, determine the length of the characteristic tool contact zones on the sample surface, and measure the inclination angle of the specimen plane. Measurement using the profilometer enables the results to be obtained with high resolution, which makes it very easy to identify the zones of tool impact on the workpiece during the cutting test and reduces measurement error values;
- The developed method allows for easier and more accurate determination of the minimum thickness of the cut layer by simplifying the geometry and preparation procedure for the test. The contact method used allows for direct and indirect determination of the minimum thickness of the cut layer;
- As the cutting speed increased, the h_min_ parameter tended to decrease. For the range of cutting speeds studied from 200 to 400 m/min, the h_min_ parameter varied from 0.93 to 1.94 µm. When planning the finishing process, the cutting speed must be increased in order to reduce the value of the removed layer of material;
- For C45 steel, an unfavorable upward trend in the h_min_ parameter was observed with increasing feed per tooth. This necessitates increasing the thickness of the cutting layer during the planning of finishing processes to ensure material separation, avoiding elastic deformation, plastic kneading, and elastic–plastic deformation with partial cutting of the workpiece material. For the studied range of tool feed speeds from 0.02 to 0.22 mm/tooth, the h_min_ parameter varied from 1.38 to 2.61 µm. When planning the finishing process, to reduce the value of the removed material layer, the cutting speed must be increased.

## Figures and Tables

**Table 2:** Properties of C45 steel [26].

Hardness (HB)	Tensile Strength (Rm)	Yield Stress (Re)	Young’s Modulus (E)
≤229	560–850 MPa	275–490 MPa	198–207 GPa

**Table 3:** Chemical composition of C45 steel, % [26].

C	Mn	Si	P	S	Cr	Ni	Mo
0.42–0.5	0.5–0.8	Max. 0.4	Max. 0.045	Max. 0.045	Max. 0.3	Max. 0.3	Max. 0.1

**Table 4:** Cutting parameters used during the experiment.

f_z_ = 0.1, mm/Tooth				v_c_ = 300, m/Min				
Lp.	v_c_, m/min	n, rpm	f_t_, mm/min	h_min_, µm	f_z_, mm/Tooth	n, rpm	f_t_, mm/min	h_min_, µm
1.	200	1247	127	1.941	0.02	1911	38	1.475
2.	220	1401	140	1.848	0.04	1911	76	2.059
3.	240	1529	153	1.485	0.06	1911	115	2.059
4.	260	1656	166	1.234	0.08	1911	153	1.386
5.	280	1783	178	1.333	0.10	1911	191	1.38
6.	300	1911	191	1.38	0.12	1911	229	2.182
7.	320	2038	204	1.553	0.14	1911	268	1.678
8.	340	2166	217	1.516	0.16	1911	306	1.86
9.	360	2293	229	1.43	0.18	1911	344	2.26
10.	380	2420	242	1.742	0.20	1911	382	2.607
11.	400	2548	255	0.929	0.22	1911	420	2.138

**Table 5:** ANOVA results for the minimum thickness of the machined layer.

Source	SS	DFs	MS	F Value	*p* Value	Percentage Contribution
Model	1.512884	4	0.378221	3.403189	0.033974	—
Constant	0.047019	1	0.047019	0.423070	0.524640	—
v_c_	0.010952	1	0.010952	0.098543	0.757640	2.14
v_c_^2^	0.024165	1	0.024165	0.217434	0.647288	4.72
f_z_	0.134267	1	0.134267	1.208119	0.287969	26.2
f_z_^2^	0.342982	1	0.342982	3.086114	0.098080	66.94
Error	1.778195	16	0.111137	—	—	0.4618
Total	3.850464	20	—	—	—	100

SS: sum of squres, DFs: degrees of freedom, MS, mean square.

## Data Availability

Data are contained within this article.
